# Phenotypic Investigation of Florfenicol Resistance and Molecular Detection of *flo*R Gene in Canine and Feline MDR Enterobacterales

**DOI:** 10.3390/vetsci11020071

**Published:** 2024-02-04

**Authors:** Marios Lysitsas, Eleutherios Triantafillou, Vassiliki Spyrou, Charalambos Billinis, George Valiakos

**Affiliations:** 1 Faculty of Veterinary Science, University of Thessaly, 431 00 Karditsa, Greece; mlysitsas@uth.gr (M.L.); billinis@uth.gr (C.B.); 2 Vet Analyseis, Private Diagnostic Laboratory, 413 35 Larissa, Greece; eltriantafil@gmail.com; 3 Department of Animal Science, University of Thessaly, 413 34 Larissa, Greece; vasilikispyrou@uth.gr

**Keywords:** florfenicol, companion animals, Enterobacterales, resistance, clinical samples, canine, feline, MDR, *flo*R, *cfr*

## Abstract

**Simple Summary:**

Florfenicol is a synthetic analog of chloramphenicol, which is used mostly in livestock animals. Its potential as an alternative agent in companion animals is strong because of its beneficial properties, such as its extensive wide spectrum and relatively increased, compared to chloramphenicol, safety during administration. However, the emergence of resistant strains can overcome this potential. The distribution of florfenicol-resistant bacteria was investigated among multidrug-resistant (MDR) Enterobacterales isolated from diagnostic samples of companion animals in Greece, which were collected throughout the country. Data regarding sample origin, type of infection, bacterial species, and resistance profiles were assembled and compared. The presence of specific florfenicol-associated antibiotic resistance genes (ARGs) was examined, and the results were interpreted in comparison with those of antibiotic susceptibility testing (AST). The results of this study indicate that the distribution of florfenicol-resistant MDR Enterobacterales in pets throughout the country is considerable (17.9%) and mainly attributed to the plasmid-mediated *flo*R gene. Thus, there is an increased risk of co-acquisition of florfenicol-specific ARGs through horizontal transfer, along with several other resistance genes. Even though the potential of florfenicol to constitute an alternative antibiotic in companion animals seems high, continuous monitoring of antibiotic resistance profiles is needed.

**Abstract:**

Florfenicol is a promising antibiotic for use in companion animals, especially as an alternative agent for infections caused by MDR bacteria. However, the emergence of resistant strains could hinder this potential. In this study, florfenicol resistance was investigated in a total of 246 MDR Enterobacterales obtained from canine and feline clinical samples in Greece over a two-year period (October 2020 to December 2022); a total of 44 (17,9%) florfenicol-resistant strains were recognized and further investigated. Most of these isolates originated from urine (41.9%) and soft tissue (37.2%) samples; *E. coli* (*n* = 14) and *Enterobacter cloacae* (*n* = 12) were the predominant species. The strains were examined for the presence of specific florfenicol-related resistance genes *floR* and *cfr.* In the majority of the isolates (31/44, 70.5%), the *floR* gene was detected, whereas none carried *cfr.* This finding creates concerns of co-acquisition of plasmid-mediated florfenicol-specific ARGs through horizontal transfer, along with several other resistance genes. The florfenicol resistance rates in MDR isolates seem relatively low but considerable for a second-line antibiotic; thus, in order to evaluate the potential of florfenicol to constitute an alternative antibiotic in companion animals, continuous monitoring of antibiotic resistance profiles is needed in order to investigate the distribution of florfenicol resistance under pressure of administration of commonly used agents.

## 1. Introduction

Florfenicol is a synthetic, fluorinated derivative of chloramphenicol, which was successfully developed in the 1980s, for usage in veterinary medicine [1,2]. It is currently used worldwide in cattle, poultry, pigs and fish [3].

This agent possesses several beneficial properties. Initially, it has an extensively wide spectrum, including most Gram-negative and Gram-positive bacteria, chlamydiae, and rickettsiae [3]. Therefore, it could constitute an alternative treating option in several cases, where available narrow-spectrum agents are not effective, mostly due to acquired antibiotic resistance. Moreover, it is not affected by several chloramphenicol-resistance mechanisms, such as the enzymatic modification by bacterial chloramphenicol acetyltransferases (CATs), as it contains a terminal fluorine instead of a hydroxyl group in its molecule [2,4]. Additionally, it possesses an obvious advantage in safety compared to chloramphenicol, due to the substitution of the nitro group (-NO_2_), which was incriminated for the drug’s serious side effects, mainly dose-unrelated aplastic anemia [2,3].

Resistance to florfenicol can be conferred by various antibiotic resistance genes (ARGs), such as *flo*R, *cfr*, *fex*A, *fex*B, and *optr*A, whereas multidrug efflux pumps have also been implicated [4]. The *Flo*R gene was first reported in an epidemic strain of *Salmonella enterica* (serovar Typhimurium) in 1999, correlated with a multiple resistance genetic locus of the emerging DT104 strain [5]. It is the predominant determinant of resistance in Gram-negative bacteria [6]. The rRNA methylase-encoding gene *cfr* was first described in a *Mammaliicoccus sciuri* isolate of bovine origin in 2000 [7]. It is commonly distributed through plasmids among bacteria of different genera and species [4].

Florfenicol’s usage is currently limited in pets. Various studies have examined its potential as a wide-spectrum alternative agent for companion animals [8,9]. Nevertheless, there are some difficulties with its administration, with the exception of otic gel formulations [10] and certain limitations must be investigated to obtain sufficient data for safe usage [11]. Therefore, further research is needed to evaluate its effectiveness in vitro at different sites of infection. However, since multidrug resistance has emerged, the armamentarium of veterinarians is limited, and evaluation of novel alternatives is necessary.

The objective of this study was to investigate the distribution and the characteristics of florfenicol-resistant Enterobacterales, with a focus on MDR isolates, since florfenicol is mostly used as a “second line” antibiotic in veterinary medicine. Moreover, we sought to identify the molecular basis of this resistance and indicate possible factors enhancing the emergence of relevant strains and concerns regarding public health.

## 2. Materials and Methods

### 2.1. Isolation and Selection of the Bacterial Strains

The bacterial strains included in this study were isolated from clinical canine and feline samples over a two-year period (October 2020 to December 2022). The samples were obtained during routine veterinary practice in veterinary hospitals throughout Greece. Isolation and biochemical identification were initially performed using conventional techniques and a commercial identification kit (Invitrogen GN-ID A, Gold Standard Diagnostics, Budapest, Hungary). Routine susceptibility testing was then performed. 

All Enterobacterales isolates demonstrating a MDR profile, according to a previously described classification [12], were collected. In cases where more than one bacterial species were obtained from a specific sample in considerable populations, all Enterobacterales were collected, regardless of the assumption about the causative agent of the infection. The strains used in this study were subsequently selected from the aforementioned group by exhibiting a phenotype resistant to florfenicol using the disc diffusion method (Kirby Bauer). The current CLSI document provides only minimum inhibitory concentration (MIC) values for Enterobacterales of swine origin [13]. As there were no specific zone diameter breakpoints for florfenicol, those provided for bovine respiratory pathogens were used (S ≥ 19 mm, I: 15–18 mm, R ≤ 14 mm). The results of the disc diffusion test were then confirmed by the MIC method (VITEK^®^2, bioMérieux, Craponne, France). All isolates were maintained in Brain Heart Infusion Broth supplemented with 20% glycerol at −80 °C.

### 2.2. Antimicrobial Susceptibility Testing

The disk diffusion method was initially performed to evaluate the susceptibility of the selected strains to the following 19 antimicrobial agents: florfenicol, chloramphenicol, ampicillin, amoxicillin + clavulanic acid, cefaclor, cefuroxime, cefoxitin, ceftazidime, piperacillin + tazobactam, amikacin, gentamicin, tobramycin, ciprofloxacin, enrofloxacin, sulfamethoxazole + trimethoprim, doxycycline, minocycline, fosfomycin, and nitrofurantoin. Both the zone diameter breakpoints and the contents of the disks, as specified by the relevant CLSI documents [13,14] (with the exception of florfenicol as previously noted), are listed in Table 1. Colonies from pure culture of each strain were added to saline in order to achieve a McFarland turbidity of 0.5 in the resulting suspension. Subsequently a sterile swab was used to inoculate a quantity of this suspension on the surface of a Mueller–Hinton agar plate. After the addition of susceptibility discs, the plates were incubated at 35 °C for 16–18 h. 

The results the AST were then confirmed with the MIC method, using VITEK^®^2 (bioMérieux, Craponne, France). The MIC for florfenicol was evaluated with the breakpoints provided by the relevant CLSI document for swine isolates (S ≤ 4, I:8, R ≥ 16) [13]. All respective breakpoints are presented in Table 1.

### 2.3. Detection of Florfenicol Resistance Genes

As noted above, various categories of ARGs related to phenicol resistance have been described in the literature [2,4,15]. In this study, we focused on florfenicol resistance and excluded all categories of genes related to chloramphenicol resistant-florfenicol susceptible phenotypes, such as chloramphenicol acetyltransferases (*CATs*) and *cml*A, *cml*B_1_ specific exporters [2,4,15]. Additionally, because bacteria of the Enterobacterales order were collected and studied, only ARGs routinely reported from these species were screened. Consequently, we excluded *cmr* and *cmx* genes, which are mainly connected to *Rhodococcus* spp. [16,17] and *Corynebacterium* spp. [18], respectively, as well as *fexA, fexB* and *optrA*. Particularly, phenicol exporter gene *fexA* is routinely correlated with Gram-positive bacteria such as *Staphylococcus* spp., *Enterococcus* spp. and *Bacillus* spp. [3,19,20,21], while *fexB* and *optrA* are strongly related to *Enterococcus* spp [4,22,23,24,25,26]. In conclusion, we examined the isolates for the presence of the *floR-specific exporter-coding* gene and the RNA methylase-coding gene *cfr,* as they are, according to the literature [2,3,4,27], common resistance determinants associated with Enterobacterales.

Whole genomic DNA was extracted from all strains, using a commercial spin column kit (IndiSpin Pathogen Kit, INDICAL BIOSCIENCE GmbH, Leipzig, Germany). All procedures were performed according to the manufacturer’s instructions. To perform PCR, previously described primers were evaluated [3]. Primers used are listed in Table 2.

For each reaction, a 25 μL mix was created per strain by adding 12.5 μL of Xpert Fast Mastermix (2X) with dye (GRiSP Research Solutions, Porto, Portugal), 2 μL (10 pmol) of each primer, 0.5 μL of bacterial DNA and 8 μL of PCR-grade water. The conditions were as follows: 95 °C for 1 min, followed by 40 cycles of 95 °C for 15 s (denaturation), 60 °C for 15 s (annealing), and 72 °C for 3 s (elongation), followed by a final extension at 72 °C for 3 min. DNA products for each gene were identified after electrophoresis in 0.5 Tris-borate-EDTA using a 1.5% agarose gel stained with ethidium bromide solution.

## 3. Results

### 3.1. Isolates, Origin of the Samples and Co-Infections

A total of 246 MDR Enterobacterales isolates were obtained from clinical samples of pets during the study period, 151 (61.4%) of which were of canine origin and 95 (38.6%) of feline. A group of 44 of them (17.9%) were resistant to florfenicol. Thirty of these isolates (68.2%) originated from canine infections, whereas the remaining 14 (31.8%) were from cats. Therefore, the resistance rate was 19.9% and 14.7% in canine and feline strains, respectively. The site of infection and distribution of the respective samples are presented in Table 3. Regarding the geographical distribution, florfenicol resistant isolates were obtained from samples collected throughout the country (Table S1). In particular, they originated in Athens (n = 18), Thessaloniki (n = 16), Volos (n = 4), Serres (n = 2), Giannitsa (n = 1), Heraklion (n = 1), Ierapetra (n = 1) and Kozani (n = 1).

Most isolates were obtained from urine and soft tissue samples. Feline samples mainly originated from cases of urinary tract infections (UTIs, 69.2%), while canine samples from soft tissue infections, followed by UTIs (46.7% and 30% respectively). Regarding soft tissue samples, the majority of them were wounds and skin lesions in dogs, while two samples from a surgical wound and a skin lesion were obtained from cats. 

### 3.2. Identity of the Isolates and Co-Current Infections

Several species were identified. *E. coli* (n = 14) and *Enterobacter cloacae* (n = 12) were the predominant isolates, while *Proteus mirabilis* (n = 7) and *Klebsiella pneumoniae* (n = 6) were obtained from variable samples. There were also detections of *Pluraribacter gergoviae* (n = 2), *Citrobacter freundii* (n = 1), *Klebsiella oxytoca* (n = 1) and *Morganella morganii* (n = 1). Several other bacterial species were co-isolated from the samples included in this study. More detailed data on canine and feline strains are listed in Table 4 and Table 5, respectively.

In 21 of the included samples (47.7%), more than one bacterial species was detected. Polymicrobial infections were detected mostly in the soft tissue samples (14/16, 87.5%), although interpretation of the results in these cases is sometimes complex and the discrimination between infection and contamination is challenging. Most of the relevant samples (13/14, 92.9%) were canine. In contrast, a single bacterial species was commonly isolated from the urine samples (16/18, 88.9%). The identified cases of polymicrobial UTIs were both felines. Two MDR Enterobacterales were identified in both cases (*E. coli* (FC12) and *Enterobacter cloacae* (FC17), *Citrobacter freundii* (FC40), and *E. coli* (florfenicol-susceptible)). Moreover, in twelve cases, two or more MDR bacteria were obtained. The co-current MDR (but florfenicol-susceptible) isolates were mostly methicillin-resistant staphylococci (*pseudintermedius* (n = 5), *aureus* (n = 2)) and Enterobacterales (*E. coli* (n = 2), *K. pneumoniae* (n = 1), *Proteus mirabilis* (n = 1)).

### 3.3. Antimicrobial Susceptibility Testing

As only MDR isolates were included in this study, high rates of resistance were unsurprisingly detected against most of the agents tested. Data regarding the resistance rates by the disc diffusion test and MIC methods are presented in Table 6 and Table 7, respectively. Detailed data for each strain are provided in Tables S1 and S2.

The results of the disc diffusion and the MIC method were mostly coincident, with a few exceptions. Regarding florfenicol, all isolates were resistant in both tests.

The majority of the strains exhibited a resistance profile against several routinely used antibiotics in veterinary medicine, such as β-lactams (ampicillin (100%), cefalexin (100%), amoxicillin − clavulanate (93.2% and 88.6%), cefaclor (97.7%), cefuroxime (93.2%)), fluoroquinolones (93.2% and 95.5%), folate pathway inhibitors (86.4%), and tetracyclines (from 68.2% to 100%). Relatively lower rates were detected for aminoglycosides, fosfomycin, and nitrofurantoin, whereas neomycin (4.5%), amikacin (13.6%) and fosfomycin (9.1%) demonstrated greater effectiveness in vitro.

### 3.4. Detection of Florfenicol Resistance Genes

**Figure 1:**
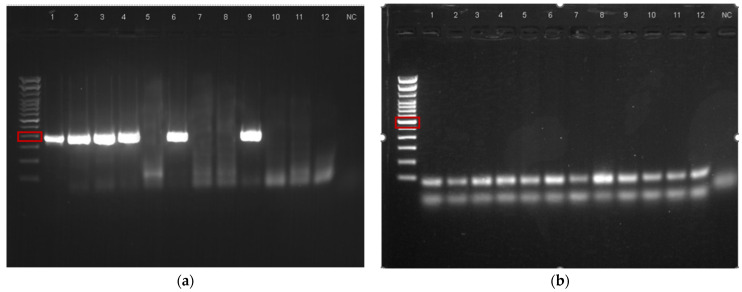
PCR gel electrophoresis images: (**a**) product of approximately 400bp size detected in isolates FC1, FC2, FC3, FC4, FC6, and FC9, indicative of *the presence of floR*; (**b**) product at 580 bp not detected, indicating the absence of *cfr* in isolates FC1 to FC12.

The *floR* gene (primers: F: 5′-ACGTTTATGCCAACCGTCCT-3′, R: 5′-CATTACAAGCGCGACAGTGG-3′) was detected in most of the isolates (31/44, 70.5%). In total, 22 of the 30 canine (73.3%) and 9 of the 14 feline (64.3%) isolates were *floR*-positive. In contrast, none of the strains carried *cfr* (Table 8, Figure 1).

Moreover, *floR*-positive bacteria exhibited a significantly limited or totally absent inhibition zone for florfenicol when tested by the disc diffusion method (Table S1). The mean inhibition zone in bacteria carrying *flo*R was approximately 7.5 mm (the total absence of inhibition zone was measured at 6 mm, which is the diameter of the discs), whereas it was significantly wider (12.5 mm) in isolates where the gene was not detected (unpaired *t*-test, *t* = 8.2911, df = 42, *p* < 0.0001). Comparable results were observed for chloramphenicol (6.9 mm for *floR*-positive and 10.6 mm for the rest) (unpaired *t*-test, *t* = 4.7952, df = 42, *p* < 0.0001). Thus, a correlation between the phenotype (inhibition zone diameter) and genotype (*floR* gene presence) was identified.

In reference to the respective MIC results, isolates FC12, FC14 and FC25 had an MIC of 16 for florfenicol, whereas all other isolates exhibited higher values (≥32). These three bacteria were all *floR*-negative. Correspondingly, the MIC for chloramphenicol for strains FC10, FC12, FC32 and FC39 was 32, while all other strains exhibited values ≥64. Only FC39 of the aforementioned group was *floR*-positive. Thus, a correlation between the presence of *floR* gene and higher MIC values is also indicated.

Regarding the geographical distribution, *floR*-positive isolates were obtained from samples collected throughout the country (Table S1). In particular, they originated in Athens (n = 17), Thessaloniki (n = 9), Volos (n = 2), Heraklion (n = 1), Ierapetra (n = 1), and Serres (n = 1).

## 4. Discussion

The results of this study demonstrated characteristics of florfenicol-resistant Enterobacterales and the respective canine and feline clinical samples in Greece. Moreover, the *floR* gene is the most prevalent molecular resistance determinant. To our knowledge, this is the first report of florfenicol-resistant Enterobacterales and the first investigation of associated ARGs in companion animals in the country. In addition, although several relevant studies have been conducted worldwide on livestock animals, data regarding pets are limited.

The resistance rate of florfenicol in MDR isolates was 17.9%, which is definitely considerable for a second-line antibacterial agent. However, in comparison with the rates documented for other classes of antibiotics, available for usage in companion animals, this percentage could be considered adequate to indicate florfenicol’s potential. Additionally, its usage entails a lower risk for human medicine than other treatment options for MDR bacteria [28]. The results of this study are in accordance with most previous reports, as relatively low percentages of resistant isolates were identified in canine and feline Enterobacterales [29,30,31,32,33,34]. Nevertheless, significant percentages of resistance were documented in a four-year surveillance study in China [35]. The prevalence of resistance in specific areas can be enhanced by antibiotic consumption in livestock animals [6]. In conclusion, even though current data about pets are limited, florfenicol demonstrated good efficacy against canine and feline isolates in most cases. Therefore, these results support the hypothesis that it could constitute an alternative “second-line” antibiotic for companion animals.

However, the presence of isolates demonstrating a florfenicol resistance profile raises concerns regarding their further distribution under the pressure of a significant limitation of available treatment options against MDR infections, which has been documented in veterinary medicine in recent decades. In addition, florfenicol resistance genes including *flo*R, which was the only one detected in this study, are regularly plasmid-encoded [2,4]. Thus, the danger of horizontal gene transfer under selection pressure is definitely not negligible, especially in cases of wide administration. Furthermore, the isolation of relevant strains from infection sites indicates the clinical importance of this issue, owing to the requirement for effective antibiotic treatment. Concludingly, even though the documented resistance rate could partially support its usage against MDR infections, this usage entails an important risk of distribution of resistance among bacterial populations.

In addition, certain limitations have to be taken into consideration regarding florfenicol’s toxicity, administration, pharmacokinetic and pharmacodynamic properties. Particularly, the risk of bone marrow suppression, anaphylactic reactions and side effects from the gastrointestinal tract are notable concerns, especially for non-topical treatment [11]. Moreover, its solubility in water is relatively low and thus the formulation of concentrated aqueous solutions in organic solvents (suitable for oral dosing) is difficult [36]. Consequently, its administration in pets is challenging with the exception of the otic gel. Further research is undoubtedly essential for obtaining sufficient data for its safe and efficient usage. The application of new technologies, like nanotechnology, could be beneficial. The use of nanoemulsions for example increased its bioavailability in pigs [36], while more types of nanostructures could be evaluated in the future to improve its effectiveness, stability and drug delivery or reduce its dosage, frequency of administration and toxicity [11].

In this study, *flo*R was the only identified gene, detected mostly in *Ε. cloacae* (n = 8), *E. coli* (n = 7), *Proteus mirabilis* (n = 7), and *Klebsiella pneumoniae* (n = 5). In relevant articles, including bacteria from pets, the *floR* gene has occasionally been detected, mainly in *E. coli* and *Proteus mirabilis* isolates [31,34,37]. However, in one study, the gene was widely disseminated, possibly through nosocomial *E. coli* strains that caused infections in dogs [38]. Its predominance in florfenicol-resistant Enterobacterales has also been demonstrated in studies on livestock animals [3,39].

The distribution of *floR* through plasmids between different species, such as *Enterobacter cloacae* and *Citrobacter freundii,* has also been highlighted [39]. Both species were identified in this study. Moreover, horizontal transfer through plasmids has been identified in isolates of both human and animal origin, and the potential of spreading between animal and human pathogens has been pointed out [6,40]. Accordingly, the susceptibility profiles documented in this study included extremely high resistance rates for specific classes of antibiotics, such as β-lactams, tetracyclines, and folate pathway inhibitors, which could be mediated by ARGs acquired through plasmids. In addition, co-localization of the respective genes has already been identified in canine gut bacteria [41]. Therefore, concerns have arisen regarding the distribution of relevant mobile genetic elements (MGEs) among canine and feline Enterobacterales, conferring a multi-resistant profile to the associated strains. The possibility that these strains will spread further under the pressure of wide administration of agents commonly used in veterinary medicine is all but negligible. Thus, surveillance measures and sparing and targeted antibiotic consumption should be priorities.

The results of the disc diffusion and the MIC test in this study were associated with the presence or absence of a *floR*-specific exporter. Positive isolates regularly exhibited an extremely limited inhibition zone or a higher MIC value, respectively, for both florfenicol and chloramphenicol, compared to negative ones. This was anticipated because this gene confers high-level phenicol resistance and is commonly correlated with high MICs in AST [3,4]. Thus, an indication of the basis of resistance is possibly provided by AST, but molecular investigation is essential for identification.

Despite the results of the susceptibility test, no ARGs (*floR* and *cfr*) were detected in a group of thirteen isolates. Therefore, the molecular basis of the florfenicol-resistant phenotypes is diverse. More mechanisms that could confer florfenicol resistance have been described, such as multidrug transporter systems, and several of them have been associated with *E. coli* [2,4]. Thus, the high percentage (7/14, 50.0%) of florfenicol-resistant *E. coli* in the aforementioned group (Table 8) could be explained by acquisition of the respective mechanisms. Molecular investigations of additional ARGs, including multidrug efflux pumps, could provide sufficient data regarding the determinants of resistance and their distribution.

This study has certain limitations. Initially, only MDR bacteria were included, since florfenicol is not commonly used in companion animals, and thus not routinely tested in the AST in cases of susceptibility to “first line” antibiotics. Even though clinical samples were examined, data regarding each case history and previous medication were not evaluated. Furthermore, only species of the Enterobacterales order were selected and examined, whereas more species are regularly implicated in phenicol-resistance, such as staphylococci, enterococci or *Acinetobacter* spp. Additionally, the interpretation regarding the causative agent of the infection in some samples (especially soft tissue ones) was difficult and thus the possibility that some strains could constitute contamination is considerable. For example, the pathogenicity of some species like *Pluralibacter gergoviae* for dogs and cats is debatable. Moreover, in vitro susceptibility was estimated using zone diameter and MIC breakpoints from bovine respiratory pathogens and swine *Salmonella enterica,* respectively. The lack of specific breakpoints for canine and feline pathogens creates doubts regarding the reliability of the AST results and their actual evaluation in clinical practice. Finally, the presence of two florfenicol-specific ARGs was investigated, whereas more genes, like multidrug efflux pumps, could be implicated in the resistance.

Nonetheless, a large number of clinical samples originating from veterinary clinics throughout the country and collected during a two-year period was included in the initial selection phase. Moreover, all isolated bacteria could constitute opportunistic pathogens for both animals and humans and also contribute to the horizontal distribution of resistance and affect the outcome of antibiotic treatment. Therefore, these results are clinically significant and highly representative of the current situation in the country.

In reference to future research perspectives, an extensive surveillance study is essential in companion animals in Greece, since current data are limited, while the risk of the distribution of MDR strains through pets is definitely significant. This study should include data regarding the medication history of the animals in order to provide comprehensive epidemiological results and the association between preceding antibiotic treatment and emerging resistance. Moreover, an extensive molecular investigation of MGEs and horizontal gene transfer could provide sufficient data regarding the acquisition of resistance to several agents, including florfenicol. Finally, the potential of florfenicol as an alternative agent in veterinary medicine, alone or in combination, should be thoroughly examined in order to obtain sufficient information about its efficacy against various types of infection in companion animals, its toxicity and possible side effects during therapy; the application of new technologies should be also investigated as it could potentially improve the pharmacodynamic or pharmacokinetic properties.

## 5. Conclusions

Florfenicol could constitute a promising alternative antibiotic for companion animals, under the pressure of the emerging multi-drug resistance. However, specific aspects need to be thoroughly investigated for completely safe and well-targeted administration. In our results, even though florfenicol resistance among MDR Enterobacterales isolates was found to be relatively low, it was mostly mediated by the plasmid-located *flo*R gene. This gene was identified in bacteria of different genera and species throughout the country. Hence, the possibility that *flo*R is co-transferred through plasmids along with ARGs against other classes of commonly used antibiotics is significant. Therefore, even though the potential of florfenicol as an alternative agent is demonstrated, the establishment of continuous monitoring of its efficacy and antibiotic resistance profiles in companion animals is also necessary.

## Figures and Tables

**Table 1:** Antibacterial agents, disc content and breakpoints used in this study [13,14].

Class	Antibacterial Agent	Disk Content (μg)	Breakpoints	
			Inhibition Zone (mm)	MIC μg/mL
Phenicols	Florfenicol	30	S: ≥19 I:15–18, R: ≤14	S: ≤4, I:8, R: ≥16
	Chloramphenicol	30	S: ≥18 I:13–17, R: ≤12	S: ≤8, I:16, R: ≥32
β-Lactams	Ampicillin	10	S: ≥17 I:14–16, R: ≤13	S: ≤8, I:16, R: ≥32
	Amoxicillin + clavulanate	20 + 10	S: ≥18 I:14–17, R: ≤13	S: ≤ 8/4, I:16/8, R: ≥32/16
	Cefalexin	-	-	S: ≤16, R: ≥32
	Cefaclor	30	S: ≥18 I:15–17, R: ≤14	-
	Cefuroxime	30	S: ≥23 I:15–22, R: ≤14	-
	Cefoxitin	30	S: ≥18 I:15–17, R: ≤14	-
	Ceftazidime	30	S: ≥21 I:18–20, R: ≤17	-
	Cefovecin	-	-	S: ≤2, I:4, R: ≥8
	Ceftiofur	-	-	S: ≤2, I:4, R: ≥8
	Cefpodoxime	-	-	S: ≤2, I:4, R: ≥8
	Piperacillin + tazobactam	100 + 10	S: ≥21 I:18–20, R: ≤17	-
	Imipenem	-	-	S: ≤1, I:2, R: ≥4
Aminoglycosides	Amikacin	30	S: ≥17 I:15–16, R: ≤14	S: ≤4, I:8, R: ≥16
	Gentamicin	10	S: ≥15 I:13–14, R: ≤12	S: ≤2, I:4, R: ≥8
	Neomycin	-	-	S: ≤8, I:16, R: ≥32
	Tobramycin	10	S: ≥15 I:13–14, R: ≤12	-
Fluoroquinolones	Ciprofloxacin	5	S: ≥26 I:22–25, R: ≤21	-
	Enrofloxacin	5	S: ≥23 I:17–22, R: ≤16	S: ≤0.5, I:1–2, R: ≥4
	Marbofloxacin	-	-	S: ≤1, I:2, R: ≥4
	Pradofloxacin	-	-	S: ≤0.25, I:0.5–1, R: ≥2
Folate Pathway Inhibitors	Trimethoprim + sulph/zole	1.25 + 23.75	S: ≥16 I:11–15, R: ≤10	S: ≤ 2/38, R: ≥ 4/76
Tetracyclines	Tetracycline	-	-	S: ≤4, I:8, R: ≥16
	Doxycycline	30	S: ≥14 I:11–13, R: ≤10	S: ≤4, I:8, R: ≥16
	Minocycline	30	S: ≥16 I:13–15, R: ≤12	-
Phosphonic acid	Fosfomycin	200	S: ≥16 I:13–15, R: ≤12	-
Nitrofurans	Nitrofurantoin	300	S: ≥17 I:15–16, R: ≤14	S ≤32, I:64, R ≥128

**Table 2:** Primers used in this study.

Gene	sequence 5′→3′	Product Length (bp)	Tm = °C	Reference
*flo*R	F: ACGTTTATGCCAACCGTCCT R: CATTACAAGCGCGACAGTGG	398	55	[3]
*cfr*	F: GGGAGGATTTAATAAATAATTTTGGAGAAACAG R: CTTATATGTTCATCGAGTATATTCATTACCTCATC	580	62	[3]

**Table 3:** Collection site and origin of the samples included in this study.

Sample	Total Samples (%)	Canine Samples (%)	Feline Samples (%)
Soft tissue	16 (37.2%)	14 (46.7%)	2 (15.4%)
Urine ^1^	18 (41.9%)	9 (30%)	9 (69.2%)
Blood culture	4 (9.3%)	3 (10%)	1 (7.7%)
Upper respiratory	2 (4.7%)	1 (3.3%)	1 (7.7%)
Bile secretion	1 (2.3%)	1 (3.3%)	0 (0%)
Ear canal	1 (2.3%)	1 (3.3%)	0 (0%)
Vaginal swab	1 (2.3%)	1 (3.3%)	0 (0%)
Total ^1^	43	30	13

^1^ FC12 (*E. coli*) and FC17 (*Enterobacter cloacae*) strains were obtained from the same cat urine sample.

**Table 4:** Canine samples: sampling site, isolate species and other bacteria detected in the same samples.

Code	Sample	FFC-Resistant Isolate	Co-Current Isolates ^1^
FC1	Soft tissue	*E. coli*	*ΜRSP* (MDR) and *Enterococcus* spp. (SDR)
FC2	Soft tissue	*E. coli*	*MRSA* (MDR) and *Pseudomonas aeruginosa* (NDR)
FC4	Soft tissue	*E. coli*	ND
FC5	Soft tissue	*E. coli*	*Proteus mirabilis* (SDR)
FC6	Ear canal	*E. coli*	*Acinetobacter baumannii* (SDR)
FC7	Soft tissue	*E. coli*	*Klebsiella pneumoniae* (MDR) and *MRSP* (MDR)
FC8	Urine	*E. coli*	ND
FC9	Soft tissue	*E. coli*	*ΜRSP* (MDR)
FC10	Blood	*E. coli*	ND
FC11	Soft tissue	*E. coli*	*P. mirabilis* (SDR) and *Enterococcus* spp. (NDR)
FC13	Tracheal secretion	*E. coli*	ND
FC16	Soft tissue	*Enterobacter cloacae*	*Staphylococcus pseudintermedius* (SDR), *P. aeruginosa* (NDR)
FC18	Soft tissue	*Pluralibacter gergoviae*	*E. coli* (SDR)
FC19	Soft tissue	*E. cloacae*	*MRSP* (MDR)
FC20	Blood	*E. cloacae*	ND
FC22	Soft tissue	*E. cloacae*	*Ε. coli* (MDR)
FC24	Urine	*E. cloacae*	ND
FC26	Soft tissue	*P. gergoviae*	*E. coli* (SDR)
FC27	Soft tissue	*E. cloacae*	*ΜRSP* (MDR)
FC28	Soft tissue	*K. pneumoniae*	*ΜRSA* (MDR)
FC29	Blood	*Klebsiella oxytoca*	*A. baumannii* (MDR)
FC30	Urine	*K. pneumoniae*	ND
FC31	Urine	*K. pneumoniae*	ND
FC33	Urine	*K. pneumoniae*	ND
FC34	Bile secretion	*E. coli*	ND
FC35	Urine	*P. mirabilis*	ND
FC36	Urine	*P. mirabilis*	ND
FC42	Urine	*K. pneumoniae*	ND
FC43	Urine	*P. mirabilis*	ND
FC44	Vaginal swab	*Morganella morganii*	*Staphylococcus aureus* (SDR)

^1^ Isolates included in this section were obtained from the same sample as the selected bacteria. MRSP: Methicillin-resistant *Staphylococcus pseudintermedius.* SDR: Single-drug resistant: The isolate that exhibits a resistant phenotype against antibiotics of one or two different classes, not counting the intrinsic resistance mechanisms of each species. MRSA: Methicillin-resistant *Staphylococcus aureus.* NDR: No drug resistance: The isolate that exhibits full susceptibility to the tested agents, with the exception of intrinsic resistance. ND: Not detected.

**Table 5:** Feline samples: sample site, isolate species and other bacteria detected in the same samples.

Code	Sample	FFC-Resistant Isolate	Co-Current Isolates
FC3	Urine	*E. coli*	ND
FC12	Urine	*E. coli*	*E. cloacae* (MDR)
FC14	Urine	*E. cloacae*	ND
FC15	Soft tissue	*E. cloacae*	*P. mirabilis* (MDR)
FC17	Urine	*E. cloacae*	*E. coli* (MDR)
FC21	Urine	*E. cloacae*	ND
FC23	Blood	*E. cloacae*	ND
FC25	Soft tissue	*E. cloacae*	ND
FC32	Urine	*K. pneumoniae*	ND
FC37	Urine	*P. mirabilis*	ND
FC38	Nasal secretion	*P. mirabilis*	*Streptococcus* spp (SDR)
FC39	Urine	*P. mirabilis*	ND
FC40	Urine	*Citrobacter freundii*	*E. coli* (MDR)
FC41	Urine	*P. mirabilis*	ND

**Table 6:** Resistance rates of the 44 isolates included in this study by the disc diffusion method.

Antibacterial Agent	Resistance Rate (n)	Resistant Rate in Dogs (n)	Resistant Rate in Cats (n)
Florfenicol	100% (44)	100% (30)	100% (14)
Chloramphenicol	90.9% (40)	93.3% (28)	85.7% (12)
Ampicillin	100% (44)	100% (30)	100% (14)
Amoxicillin + clavulanate	93.2% (41)	90.0% (27)	100% (14)
Cefaclor	97.7% (43)	96.7%(29)	100% (14)
Cefuroxime	93.2% (41)	90.0% (27)	100% (14)
Cefoxitin	68.2% (30)	70.0% (21)	64.3% (9)
Ceftazidime	45.5% (20)	40.0% (12)	57.1% (8)
Piperacillin + tazobactam	34.1% (15)	30.0% (9)	42.9% (6)
Amikacin	13.6% (6)	20.0% (6)	0.0% (0)
Gentamicin	38.6% (17)	40.0% (12)	35.7% (5)
Tobramycin	43.2% (19)	43.3% (13)	42.9% (6)
Enrofloxacin	93.2% (41)	90.0% (27)	100% (14)
Ciprofloxacin	93.2% (41)	90.0% (27)	100% (14)
Sulph/zole + trimethoprim	86.4% (38)	86.7% (26)	85.7% (12)
Doxycycline	90.9% (40)	90.0% (27)	92.5% (13)
Minocycline	63.6% (28)	60.0% (18)	71.4% (10)
Fosfomycin	9.1% (4)	13.3% (4)	0.0% (0)
Nitrofurantoin	40.1% (18)	33.3% (10)	57.1% (8)

**Table 7:** Resistance rates of the 44 isolates included in this study by the MIC method.

Antibacterial Agent	Resistance Rate (n)	Resistant Rate in Dogs (n)	Resistant Rate in Cats (n)
Florfenicol	100% (44)	100% (30)	100% (14)
Chloramphenicol	100% (44)	100% (30)	100% (14)
Ampicillin ^1^	100% (29)	100% (22)	100% (7)
Amoxicillin + clavulanate	88.6% (39)	90.0% (27)	85.7% (12)
Cefalexin ^1^	100% (37)	100%(27)	100% (10)
Cefpodoxime ^1^	81.4% (35)	79.3% (23)	85.7% (12)
Cefovecin ^1^	79.1% (34)	75.9% (22)	85.7% (12)
Ceftiofur	70.5% (31)	66.7% (20)	78.6% (11)
Imipenem	31.8% (14)	26.7% (8)	42.9% (6)
Amikacin	13.6% (6)	16.7% (5)	7.1% (1)
Gentamicin	40.1% (18)	43.3% (13)	35.7% (5)
Neomycin	4.5% (2)	6.7% (2)	0.0% (0)
Enrofloxacin	95.5% (42)	93.3% (28)	100% (14)
Marbofloxacin	95.5% (42)	93.3% (28)	100% (14)
Pradofloxacin	95.5% (42)	93.3% (28)	100% (14)
Sulph/zole + trimethoprim	86.4% (38)	86.7% (26)	85.7% (12)
Tetracycline	100% (44)	100% (30)	100% (14)
Doxycycline	97.7% (43)	100% (30)	92.9% (13)
Nitrofurantoin	27.3% (12)	23.3% (7)	35.7% (5)

^1^ The rates are presented only for the number of strains tested for each agent by VITEK 2 (Table S2).

**Table 8:** Distribution of *flo*R-positive bacteria.

Bacterial Species	*flo*R-Positive Isolates	*flo*R-Negative Isolates
*C. freundii*	FC40	-
*E. cloacae*	FC16, FC17, FC19, FC20, FC21, FC22, FC23, FC24	FC14, FC15, FC25, FC27
*E. coli*	FC1, FC2, FC3, FC4, FC6, FC9, FC34	FC5, FC7, FC8, FC10, FC11, FC12, FC13
*K. oxytoca*	FC29	*-*
*K. pneumoniae*	FC28, FC30, FC31, FC33, FC42	FC32
*M. morganii*	FC44	-
*P. gergoviae*	FC18	FC26
*P. mirabilis*	FC35, FC36, FC37, FC38, FC39, FC41, FC43	*-*

## Data Availability

The data presented in this study are available in this article.

## Supplementary Materials

The following supporting information can be downloaded at: https://www.mdpi.com/article/10.3390/vetsci11020071/s1, Table S1: Detailed data of florfenicol-resistant isolates, Table S2: Results of MIC testing.
